# The mitochondrial carrier pathway transports non-canonical substrates with an odd number of transmembrane segments

**DOI:** 10.1186/s12915-019-0733-6

**Published:** 2020-01-06

**Authors:** Heike Rampelt, Iva Sucec, Beate Bersch, Patrick Horten, Inge Perschil, Jean-Claude Martinou, Martin van der Laan, Nils Wiedemann, Paul Schanda, Nikolaus Pfanner

**Affiliations:** 1 Institute of Biochemistry and Molecular Biology, ZBMZ, Faculty of Medicine, University of Freiburg, 79104 Freiburg, Germany; 2 CIBSS Centre for Integrative Biological Signalling Studies, University of Freiburg, 79104 Freiburg, Germany; 3 Institut de Biologie Structurale (IBS), Univ. Grenoble Alpes, CEA, CNRS, 38000 Grenoble, France; 4 Faculty of Biology, University of Freiburg, 79104 Freiburg, Germany; 5 Department of Cell Biology, University of Geneva, Genève 4, Switzerland; 6 Medical Biochemistry and Molecular Biology, Center for Molecular Signaling, PZMS, Saarland University, 66421 Homburg, Germany; 7 BIOSS Centre for Biological Signalling Studies, University of Freiburg, 79104 Freiburg, Germany

**Keywords:** Mitochondrial pyruvate carrier, MPC, Mitochondrial protein biogenesis, Protein import, TIM22 complex, Tim9, Tim10, TIM23 complex

## Abstract

**Background:**

The mitochondrial pyruvate carrier (MPC) plays a central role in energy metabolism by transporting pyruvate across the inner mitochondrial membrane. Its heterodimeric composition and homology to SWEET and semiSWEET transporters set the MPC apart from the canonical mitochondrial carrier family (named MCF or SLC25). The import of the canonical carriers is mediated by the carrier translocase of the inner membrane (TIM22) pathway and is dependent on their structure, which features an even number of transmembrane segments and both termini in the intermembrane space. The import pathway of MPC proteins has not been elucidated. The odd number of transmembrane segments and positioning of the N-terminus in the matrix argues against an import via the TIM22 carrier pathway but favors an import via the flexible presequence pathway.

**Results:**

Here, we systematically analyzed the import pathways of Mpc2 and Mpc3 and report that, contrary to an expected import via the flexible presequence pathway, yeast MPC proteins with an odd number of transmembrane segments and matrix-exposed N-terminus are imported by the carrier pathway, using the receptor Tom70, small TIM chaperones, and the TIM22 complex. The TIM9·10 complex chaperones MPC proteins through the mitochondrial intermembrane space using conserved hydrophobic motifs that are also required for the interaction with canonical carrier proteins.

**Conclusions:**

The carrier pathway can import paired and non-paired transmembrane helices and translocate N-termini to either side of the mitochondrial inner membrane, revealing an unexpected versatility of the mitochondrial import pathway for non-cleavable inner membrane proteins.

## Background

Despite its crucial role in physiology, the molecular identity of the mitochondrial pyruvate carrier (MPC) was uncovered only in recent years [1, 2]. MPC enables transport of pyruvate into the mitochondrial matrix for oxidative metabolism via pyruvate dehydrogenase and the tricarboxylic acid cycle. Due to this central position in energy metabolism, the MPC plays a crucial role in metabolic switches between glycolytic and respiratory growth and affects cancer stemness [3–5]. The functional unit of the MPC is an inner membrane-integrated heterodimer consisting of MPC1 and MPC2 in mammals and of Mpc1 with either Mpc2 or Mpc3 in yeast [1, 2, 6, 7].

**Fig. 1:**
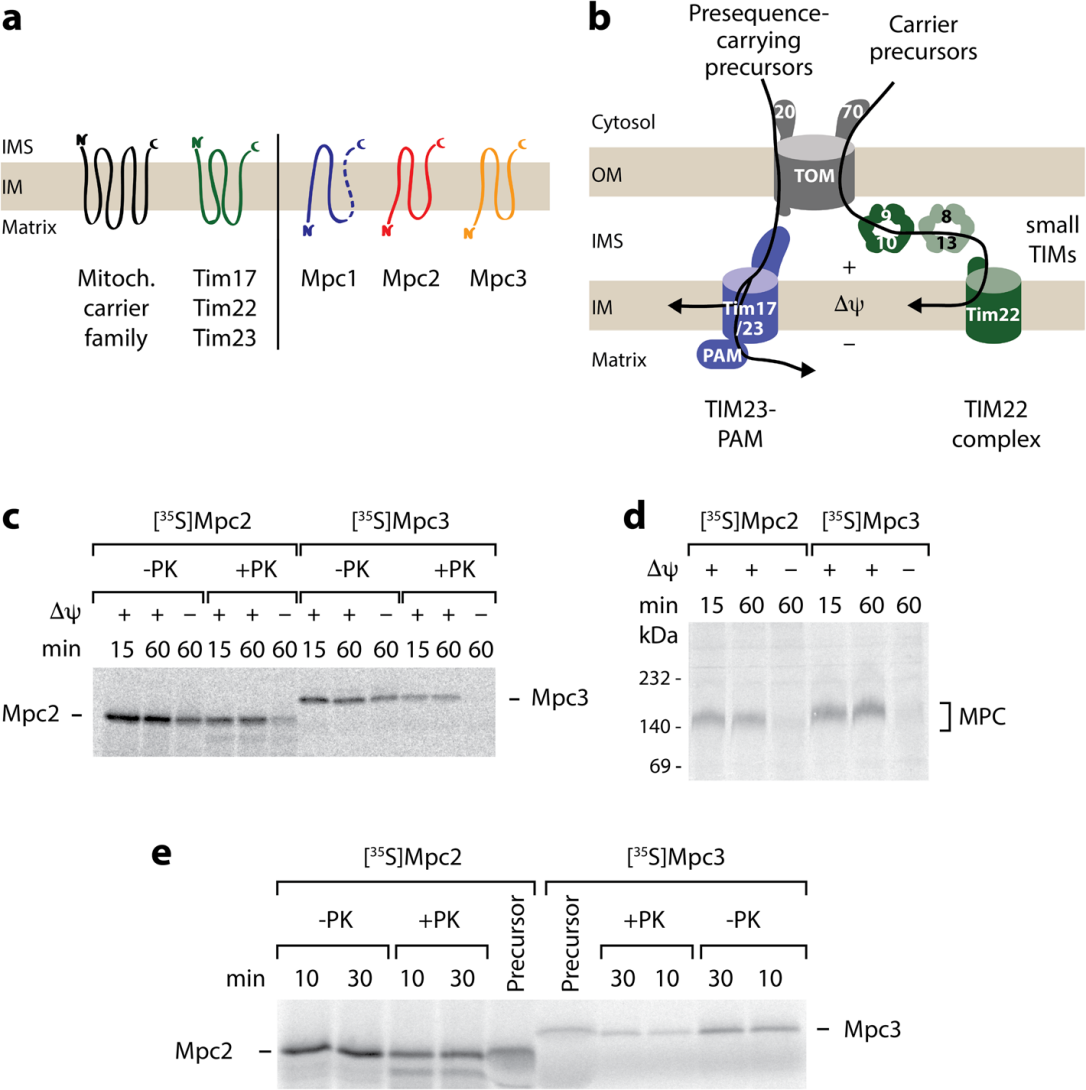
Import of MPC precursors into the mitochondria. **a** Membrane topology of substrates of the carrier translocase TIM22 in the inner mitochondrial membrane (IM). Left, all TIM22 substrates known so far possess a uniform topology with an even number of transmembrane segments and both termini facing the intermembrane space (IMS): canonical mitochondrial carriers (black) and translocase components (green). Right, the mitochondrial pyruvate carrier subunits Mpc2 and Mpc3 possess an odd number of transmembrane segments and expose the N-terminus to the matrix. The N-terminus of Mpc1 is also located in the matrix, Mpc1 likely possesses three transmembrane segments like Mpc2/3. **b** Overview of the presequence (TIM23) pathway and the carrier (TIM22) pathway to the IM. Precursors with N-terminal presequence are recognized by the receptor Tom20, translocated through the TOM complex, and transferred to TIM23 for sorting to the IM or matrix. Precursors of the mitochondrial carrier family are recognized by the receptor Tom70, translocated through TOM, and handed over to small TIM chaperones in the IMS (TIM9·10, TIM8·13); the precursors are inserted into the IM by the TIM22 complex. Δψ, membrane potential across the IM; PAM, presequence translocase-associated motor. **c** Mpc2 and Mpc3 precursors radiolabeled with [^35^S] methionine were imported at 25 °C into isolated yeast wild-type mitochondria for the indicated periods. Non-imported precursors were degraded with proteinase K (PK) where indicated; the mitochondria were analyzed by SDS-PAGE and autoradiography. **d** Mpc2 and Mpc3 import and assembly into a native complex is Δψ-dependent. Radiolabeled Mpc2 and Mpc3 precursors were imported as in **c**; mitochondria were PK-treated and analyzed by BN-PAGE and autoradiography. **e** Mpc2 and Mpc3 are not proteolytically processed upon import into mitochondria. Mpc2 and Mpc3 were imported into mitochondria as in **c**. The reactions were analyzed by SDS-PAGE and autoradiography. For comparison, 20% of reticulocyte lysate (precursor) used in the import reactions were included

The inner mitochondrial membrane harbors a multitude of carrier proteins that belong to the mitochondrial carrier family (termed MCF or SLC25 for solute carrier family 25) and transport nucleotides, amino acids, and other metabolites across the inner membrane. These canonical, well-studied carrier proteins are characterized by three structural modules, each consisting of two transmembrane helices with a connecting matrix loop, and expose both termini of the polypeptide chain to the intermembrane space (Fig. 1a) [8–10]. MPC proteins do not belong to the established mitochondrial carrier family but are related to the SWEET (sugars will eventually be exported transporter) and semiSWEET sugar transporters that function as two triple-helix bundles [11, 12]. In contrast to the canonical carriers with six transmembrane helices, Mpc2 and Mpc3 were shown to contain three transmembrane helices with the N-terminus facing the matrix, based on the accessibility to protease or to thiol labeling (Fig. 1a) [6, 7]. The N-terminus of Mpc1 faces the matrix; its exact number of transmembrane segments has not been defined as biochemical approaches suggested the presence of two transmembrane segments, whereas a recent homology analysis indicated that Mpc1 displays a similar topology as Mpc2 and Mpc3 [6, 7]. The active MPC complexes are heterodimers; Mpc1-Mpc3 promotes pyruvate transport during respiratory growth, whereas Mpc1-Mpc2 functions during fermentable growth [6, 7, 13].

The import pathway of canonical carrier precursors from the cytosol to the carrier translocase of the inner mitochondrial membrane (TIM22) has been well established (Fig. 1b) [14–16]. After recognition of internal, non-cleavable signals by the receptor Tom70 of the translocase of the outer membrane (TOM) [17–19], carrier precursors pass through the TOM channel into the intermembrane space. There, the hydrophobic precursors are bound by small TIM chaperones and are transferred to the TIM22 complex for membrane potential (Δψ)-dependent insertion into the inner membrane (Fig. 1b) [15, 20–24]. In contrast to the highly versatile presequence translocase of the inner membrane (TIM23) that handles a large variety of precursor proteins, including cleavable and non-cleavable matrix and inner membrane proteins with differing topologies, the carrier translocase TIM22 is thought to have a narrow, well-defined substrate repertoire (Fig. 1a, b). The only known physiological substrates of the TIM22 pathway are the mitochondrial carriers with 6 transmembrane segments (> 30 members in fungi and > 50 members in mammals [9]) and the translocase components Tim17, Tim22, and Tim23 with 4 transmembrane segments, all sharing the same topology with both termini facing the intermembrane space (Fig. 1a) [14, 15]. Precursors imported by the carrier pathway are bound and transported by the TOM complex in a modular fashion with pairs of transmembrane helices being translocated [14, 18, 19, 23, 25]. Binding to the small TIM chaperones also takes place in a modular fashion [26]. Mutational studies with truncated carrier precursors indicated that the cooperation of the 2-helix modules is crucial for import and assembly via the carrier pathway [19, 27–29]. Truncated carrier precursors with 4 or less transmembrane segments were even mistargeted via the TIM23 complex into the matrix or remained in the intermembrane space [28, 29]. A remarkable exception in the carrier family is Ugo1 that contains an odd number of transmembrane segments (3). Indeed, Ugo1 is not imported by the TIM22 pathway but is an integral component of the mitochondrial outer membrane [30–33]. The findings reported so far thus strongly support the model of strict substrate selectivity of the TIM22 pathway.

The biogenesis pathway of MPC proteins from their synthesis in the cytosol to their mature destination in the inner membrane has not been elucidated. The odd number of transmembrane segments and positioning of the N-terminus in the matrix argues against an import via the TIM22 carrier pathway but favors an import via the flexible presequence pathway. Here, we systematically analyzed the import pathways of Mpc2 and Mpc3 and unexpectedly observed a clear dependence on the carrier import pathway, including the receptor Tom70, TIM chaperones, and TIM22 complex, but not on the presequence pathway. These findings substantially expand the substrate spectrum and translocation flexibility of the mitochondrial carrier pathway.

## Results

### Targeting and Δψ-dependent import of MPC precursors into mitochondria

We synthesized and radiolabeled the precursors of Mpc2 and Mpc3 in a cell-free system and imported them into isolated yeast wild-type mitochondria. The precursors were transported to a protease-protected location (Fig. 1c) and assembled into a complex migrating at ~ 150 kDa in blue native gel electrophoresis (Fig. 1d) like the mature assembled MPC dimers detected by Western blotting (Additional file 1: Figure S1a-d) [1, 6]. The relatively slow migration of the ~ 30 kDa MPC dimers on blue native electrophoresis is likely due to considerable amounts of lipids and detergent bound to MPC, similar to observations with other small membrane proteins [7, 26, 34, 35]. In the absence of a membrane potential Δψ, the transport to a protease-protected location was impaired and the assembly into the ~ 150 kDa complex was blocked (Fig. 1c, d), demonstrating that Δψ across the inner membrane was required for the biogenesis of the MPC proteins in line with the inner membrane localization of mature MPC. The strong Δψ dependence of the formation of the 150 kDa MPC complex upon importing radiolabeled precursors provided an efficient assay for studying import and assembly of Mpc2 and Mpc3 *in organello*. The imported proteins (+Δψ) showed the identical SDS gel mobility as the non-imported precursors (−Δψ) and the precursors synthesized in the cell-free system (Fig. 1c, e), indicating that neither Mpc2 nor Mpc3 carried a cleavable presequence, in agreement with a systematic proteomic study that did not detect a cleavable presequence in Mpc3 (termed Fmp43 before the assignment as MPC subunit) [36].

**Fig. 2:**
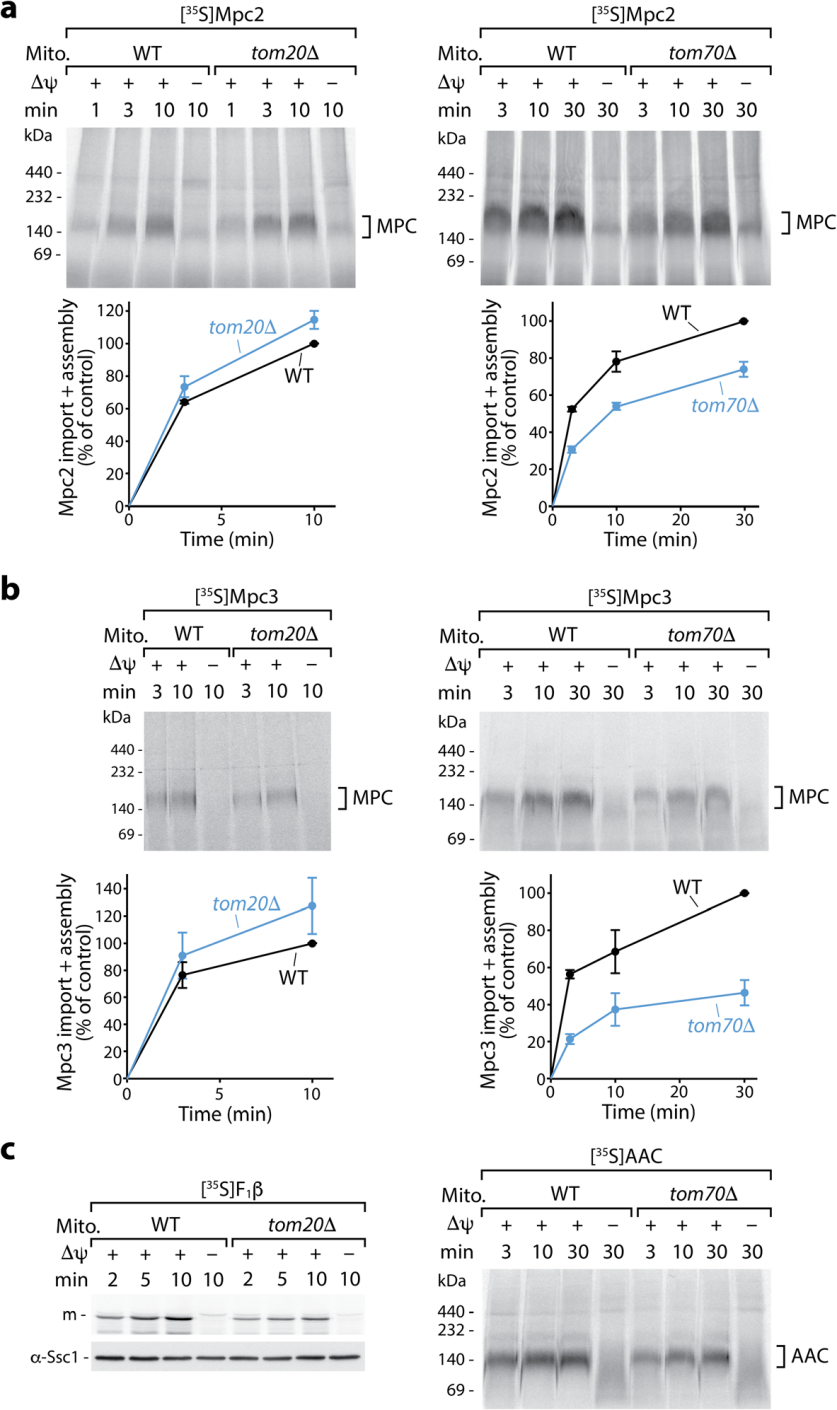
Import of Mpc2 and Mpc3 precursors occurs via the receptor Tom70, not Tom20. Radiolabeled Mpc2 (**a**) and Mpc3 (**b**) were imported at 25 °C into mitochondria from wild-type (WT), *tom20*Δ, or *tom70*Δ yeast strains and analyzed as described in Fig. 1d. **a**, **b** (lower panels) Quantification of import and assembly efficiency; the efficiency into WT mitochondria upon the longest import period was set to 100% (control); *n* = 3 except Mpc2 import into *tom70*Δ: *n* = 4; error bars: SEM. As controls, the matrix-targeted precursor of F_1_β was imported into *tom20*Δ mitochondria (**c**, left panel, with α-Ssc1 immunodecoration as a loading control), and the carrier protein AAC was imported into *tom70*Δ mitochondria (**c**, right panel). In all experiments, non-imported precursors were degraded with proteinase K. m, mature form

Precursor proteins imported via the presequence pathway are typically recognized by the receptor Tom20, whereas canonical carrier precursors are recognized by Tom70 [17, 35, 37–42]. Import and assembly of Mpc2 and Mpc3 into *tom20*Δ mitochondria were not inhibited, but even slightly better than that into wild-type mitochondria, whereas import of the presequence pathway substrate F_1_-ATPase subunit β (F_1_β) was inhibited in the mutant mitochondria as expected (Fig. 2a–c, Additional file 2: Figure S2a-c). When Mpc2 or Mpc3 were imported into the mitochondria lacking Tom70, however, we observed a reduction of import and assembly similar to that observed for the ADP/ATP carrier (AAC) (Fig. 2a–c, Additional file 2: Figure S2d-g). The individual TOM receptors do not exclusively recognize one defined substrate class but possess an overlapping specificity [37, 43, 44]. In particular, precursors with N-terminal presequences recognized by Tom20 can contain additional internal targeting signals that interact with Tom70, and thus, these precursors employ both receptors [43–45]. The selective dependence of Mpc2 and Mpc3 on Tom70 and not on Tom20 (Fig. 2a, b, Additional file 2: Figure S2 g), however, does not fit to the typical receptor dependence of preproteins with N-terminal targeting signals but to that of the mitochondrial carrier family MCF/SLC25.

### MPC precursors are imported via the TIM22 complex and not the TIM23 complex

To directly determine whether the TIM22 complex or the TIM23 complex is responsible for membrane insertion of Mpc2 and Mpc3, we imported the precursor proteins into mitochondria which were isolated from yeast mutants that specifically affect one of the translocases.

**Fig. 3:**
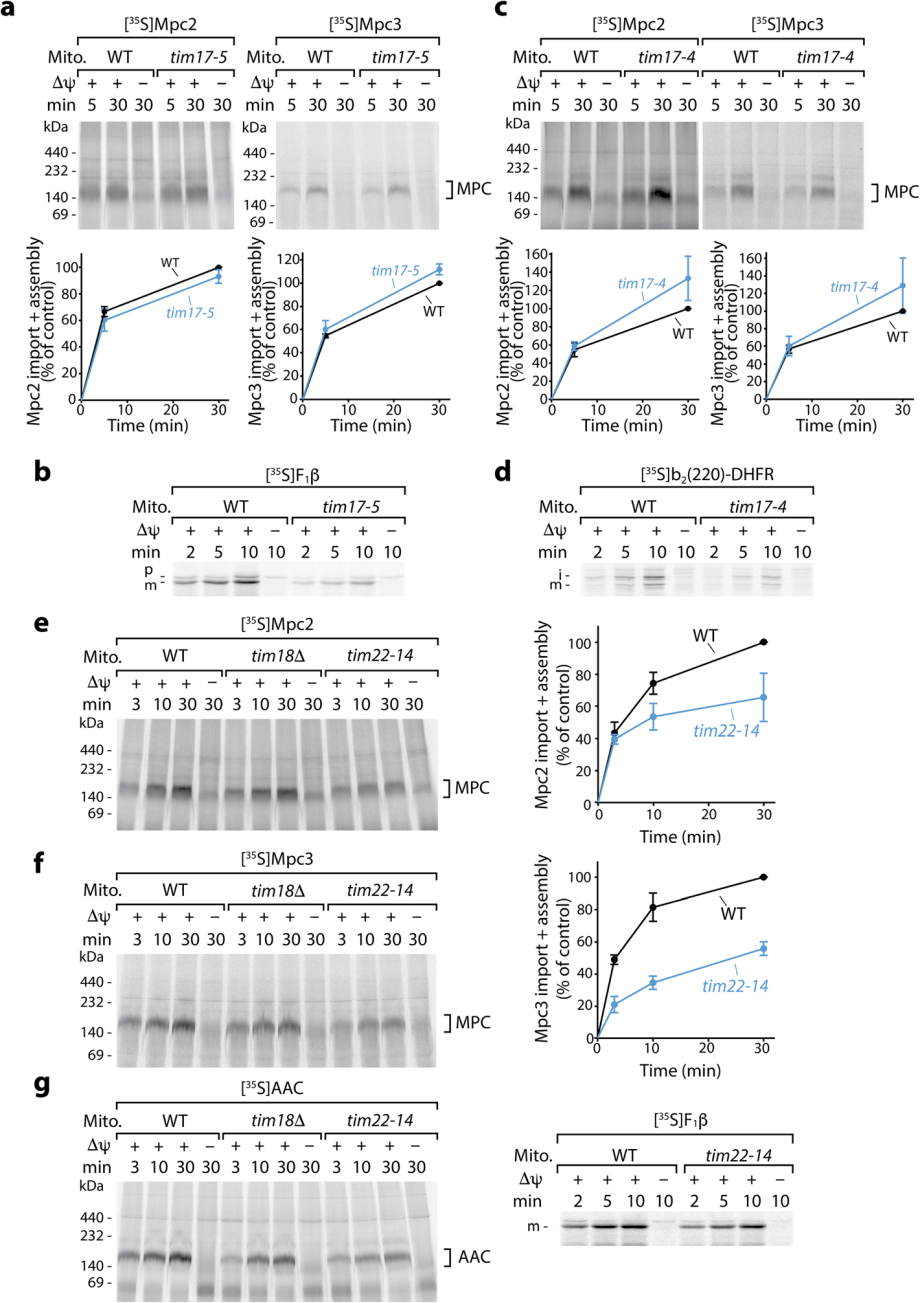
Mpc2 and Mpc3 are imported by TIM22 and are independent of TIM23. **a** Wild-type (WT) and *tim17-5* mitochondria, which display a specific defect in TIM23-mediated matrix import [46, 47], were heat-shocked for 10 min at 37 °C prior to import of radiolabeled Mpc2 or Mpc3 at 25 °C. Samples were analyzed by BN-PAGE and autoradiography. Quantification of import and assembly efficiency; the efficiency into WT mitochondria after 30 min was set to 100% (control), *n* = 3; error bars: SEM. **b** As a control, the matrix protein F_1_β was imported into heat-shocked WT and *tim17-5* mitochondria. Samples were analyzed by SDS-PAGE and autoradiography. p, precursor; m, mature form. **c** Mpc2 and Mpc3 were imported at 25 °C into heat-shocked WT mitochondria and *tim17-4* mitochondria that display a defect in TIM23-mediated sorting into the inner membrane [46, 47]. Samples were analyzed and quantitated as in **a**; *n* = 3; error bars: SEM. **d** As a control, the IM sorting substrate b_2_(220)-DHFR was imported into heat-shocked WT and *tim17-4* mitochondria. Samples were analyzed by SDS-PAGE and autoradiography. i, intermediate form; m, mature form. **e** Mpc2 was imported at 25 °C into mitochondria from WT and TIM22-specific yeast mutant strains, *tim18*Δ or *tim22-14*, and analyzed by BN-PAGE and autoradiography. Quantification of import and assembly efficiency as in **a**; *n* = 3; error bars: SEM. **f** Mpc3 was imported at 25 °C into mitochondria from WT, *tim18*Δ and *tim22-14* strains as in **e**. Quantification of import and assembly efficiency as in **a**; *n* = 3; error bars: SEM. **g** The model carrier substrate AAC was imported at 25 °C into *tim18*Δ and *tim22-14* mitochondria (left panel) and analyzed as the Mpc2/Mpc3 import reactions. As a control, the matrix-targeted precursor of F_1_β was imported into these mitochondria (right panel) and analyzed by SDS-PAGE and autoradiography. m, mature form. In all experiments, non-imported precursors were degraded with PK

To date, all imported mitochondrial proteins that expose the N-terminus to the matrix have been found to be transported by the TIM23 pathway [14, 15]. Since the MPC proteins also expose their N-termini to the matrix, we analyzed the dependence on the TIM23 machinery. The yeast mutants *tim17-5* and *tim17-4* selectively impair TIM23-mediated matrix import or lateral sorting of cleavable preproteins into the inner membrane, respectively, without disturbing the inner membrane potential and the canonical carrier import [46, 47]. Import and assembly of Mpc2 and Mpc3, however, were not inhibited in *tim17-5* mitochondria after an in vitro heat shock at 37 °C (Fig. 3a, Additional file 3: Figure S3a; the corresponding wild-type mitochondria were subjected to the same heat shock conditions), whereas import of the TIM23-dependent matrix protein F_1_β was considerably impaired (Fig. 3b). Unexpectedly, heat-shocked *tim17-4* mitochondria, which were impaired in the inner membrane sorting of the TIM23 model substrate b_2_(220)-DHFR [46, 47], efficiently imported and assembled Mpc2 and Mpc3 in a Δψ-dependent manner (Fig. 3c, d; Additional file 3: Figure S3b), indicating that the MPC proteins are not imported by the presequence pathway.

The lack of the non-essential subunit Tim18 of the TIM22 complex only mildly affected the import and assembly of Mpc2, Mpc3, and AAC (Fig. 3e–g, Additional file 3: Figure S3c) and thus did not provide an answer on the translocase dependence. Therefore, we used the yeast temperature-sensitive mutant *tim22-14* of the essential translocase subunit Tim22 at a permissive temperature [48] (Additional file 3: Figure S3d). The mutant mitochondria are disturbed in the assembly of the carrier translocase TIM22 [48]. Despite mildly reduced levels of the TIM22 substrate Tim23 (Additional file 3: Figure S3d), neither the presequence import pathway (Fig. 3g, right panel) nor the inner membrane potential is impaired [48]. Import and assembly of Mpc2 and Mpc3, however, were partially reduced in *tim22-14* mitochondria, like import and assembly of the canonical substrate AAC (Fig. 3e–g, Additional file 3: Figure S3c, e, f), supporting the view that the MPC proteins use the carrier import pathway.

Taken together, we conclude that the two MPC proteins are imported via the TIM22 pathway into the inner membrane despite their non-canonical carrier topology and their odd number of transmembrane segments.

### Import of MPC precursors involves small TIM chaperones of the intermembrane space

Canonical carrier proteins with their six hydrophobic transmembrane segments rely on chaperoning by the small TIM proteins during their transit through the aqueous intermembrane space, providing a strict difference to the presequence import pathway where precursors are directly transferred from the TOM complex to the TIM23 complex [20–23, 26, 45, 46, 49–53]. Carrier precursors are preferentially bound by the essential Tim9-Tim10 complex (TIM9·10), while the alternative Tim8-Tim13 complex (TIM8·13) provides some redundancy and, together with TIM9·10, promotes the import of β-barrel precursors to the outer membrane [26, 54]. The association of carrier precursors with the TIM22 complex is accomplished via a membrane-bound module of TIM22 comprising Tim9, Tim10, and Tim12 [20, 22, 24, 55].

**Fig. 4:**
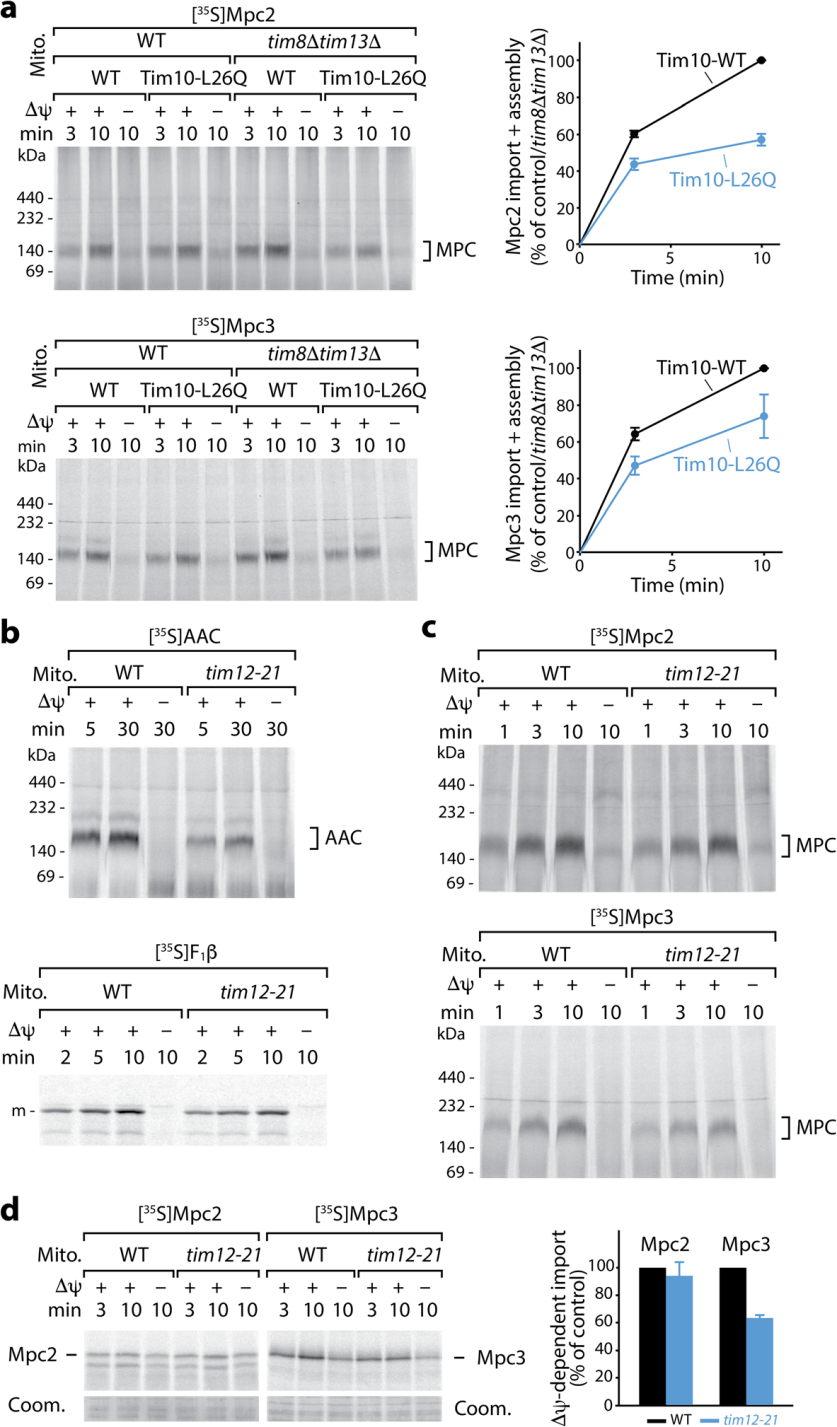
Mpc2 and Mpc3 import depends on small TIM chaperones. **a** Radiolabeled Mpc2 and Mpc3 were imported at 25 °C into wild-type (WT) mitochondria, mitochondria with the mutant form Tim10-L26Q, mitochondria lacking Tim8 and Tim13, or mitochondria affected in Tim10, Tim8, and Tim13 [26]. Samples were analyzed by BN-PAGE and autoradiography. Quantification of import and assembly efficiency; the efficiency into Tim10-WT/*tim8*Δ*tim13*Δ mitochondria after 10 min was set to 100% (control); *n* = 3 for Mpc2 import, *n* = 4 for Mpc3 import; error bars: SEM. **b** AAC (upper panel) and F_1_β (lower panel) were imported at 30 °C into wild-type or *tim12-21* mutant mitochondria, followed by BN-PAGE (AAC) or SDS-PAGE (F_1_β) analysis and autoradiography. m, mature form. **c** Mpc2 (upper panel) and Mpc3 (lower panel) were imported at 30 °C into wild-type or *tim12-21* mutant mitochondria and analyzed by BN-PAGE and autoradiography. **d** Mpc2 or Mpc3 were imported at 30 °C into wild-type or *tim12-21* mutant mitochondria. Mitoplasts were generated by hypo-osmotic swelling, treated with proteinase K, and analyzed by SDS-PAGE and autoradiography (upper panel) or Coomassie Blue R-250 staining (Coom.) as a loading control (lower panel). Quantification (right panel) of membrane potential (Δψ)-dependent import yield after 10 min relative to the WT control; *n* = 3; error bars: SEM. In all experiments, non-imported precursors were degraded with proteinase K

The model of MPC import via the canonical carrier import pathway implies that MPC precursors should depend on small TIM chaperones for crossing the intermembrane space. We thus asked if any of the TIM chaperones participated in the import of Mpc2 and Mpc3. We used a yeast mutant of the TIM9·10 complex containing an amino acid replacement in the chaperone motif of the essential Tim10 protein, resulting in a temperature-sensitive growth defect. Tim10-L26Q mutant mitochondria are delayed in the import of canonical carrier proteins and the four-transmembrane substrate Tim23 under permissive conditions, whereas Δψ and the presequence pathway are not affected [26]. Import and assembly of Mpc2 and Mpc3 into the Tim10-L26Q mitochondria at permissive temperature were reduced both in the presence and in the absence of TIM8·13, similarly to the biogenesis of AAC (Fig. 4a, Additional file 4: Figure S4a-c). The lack of TIM8·13 alone did not impede Mpc2/3 import (Fig. 4a). The steady-state levels of Mpc1 and Mpc3 were reduced in the Tim10-L26Q mutant strains, but not in the *tim8*Δ*tim13*Δ strain, similarly to the levels of the canonical carrier protein Yhm2 (Additional file 4: Figure S4d). The increased levels of Mpc2 in the Tim10-L26Q mutant strains are likely due to the decreased levels of Mpc1 as the lack of Mpc1 leads to a strong induction of Mpc2 levels (Additional file 1: Figure S1a-f) [1]. A preferential dependence on the essential TIM9·10 chaperone and a backup function of the non-essential TIM8·13 chaperone are consistent with the import behavior of carrier pathway substrates like AAC and Tim23 and distinguish Mpc2/3 from the import characteristics of β-barrel precursors that typically use both TIM9·10 and TIM8·13 [26]. To address a possible requirement for inner membrane-bound Tim12, we tested the import of the MPC precursors into mitochondria from the temperature-sensitive *tim12-21* mutant, employing the elevated temperature of 30 °C. The *tim12-21* mutant mitochondria were impaired in the carrier pathway (AAC), but not in the presequence pathway (F_1_β) (Fig. 4b) [55]. Import and assembly of Mpc2 were not significantly diminished in the mutant mitochondria, whereas Mpc3 was partially affected (Fig. 4c, d) and the import of AAC was more strongly reduced (Fig. 4b). These results suggest that the biogenesis of Mpc2/3 involves small TIM proteins, in particular the major soluble TIM chaperone, the TIM9·10 complex.

**Fig. 5:**
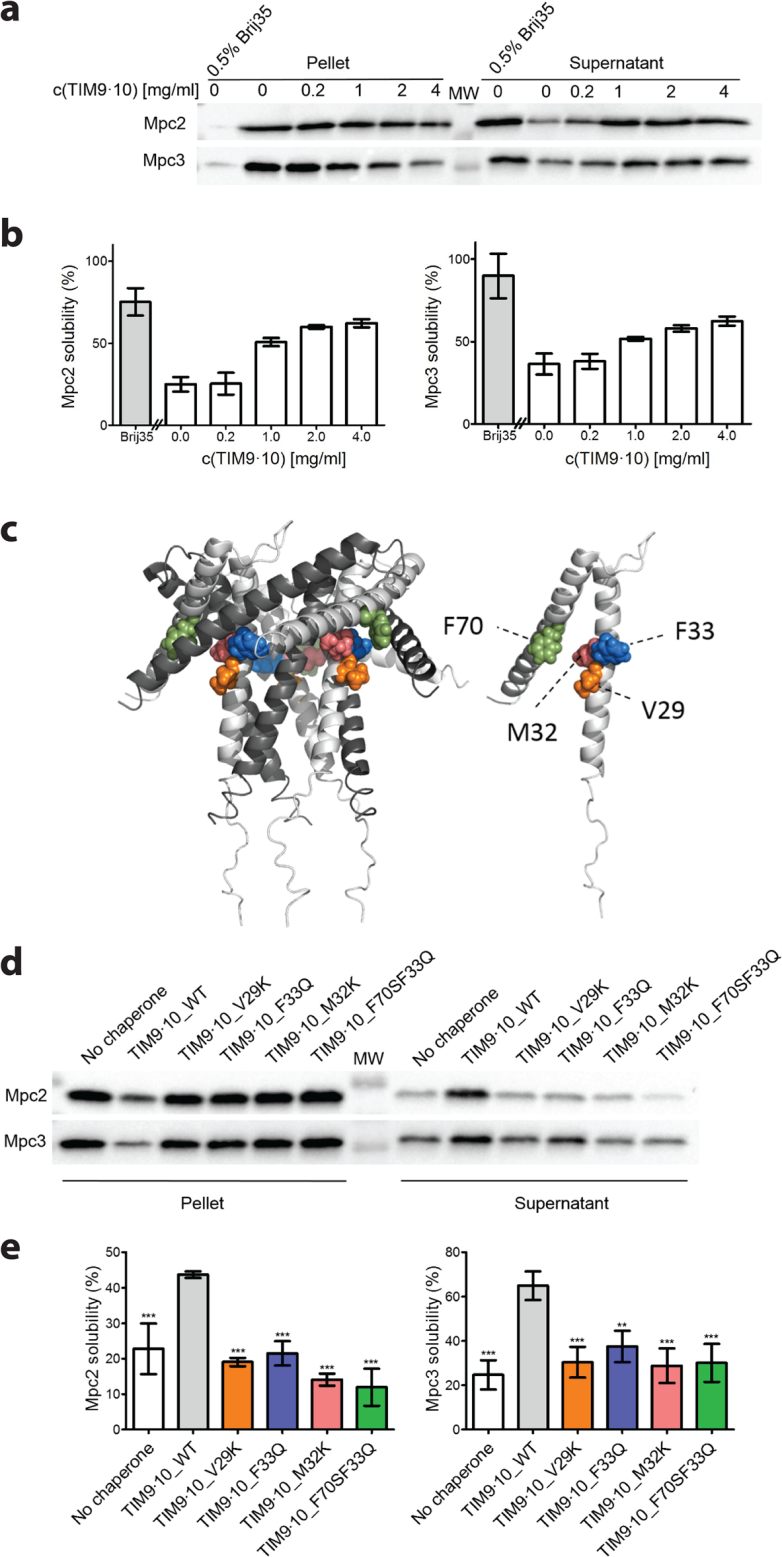
Interaction of Mpc2 and Mpc3 with the TIM9·10 chaperone in vitro. **a** Cell-free reaction mixtures producing Mpc2 (upper panel) or Mpc3 (lower panel) were supplemented with detergent (Brij35) or different concentrations of recombinantly produced TIM9·10 complex. Immunoblot of the soluble (supernatant) and insoluble (pellet) fractions of the reaction mixtures. **b** Mpc2 and Mpc3 solubility quantification. In the presence of detergent (absence of TIM9·10), both Mpc2 and Mpc3 were largely found in the soluble fraction. In the absence of detergent and chaperone, the majority of Mpc2 and Mpc3 was found in the insoluble fraction. Increasing the concentration of TIM9·10 complex in the cell-free reaction mixture resulted in increased solubility of Mpc2 and Mpc3; *n* = 4–5 for Mpc2; *n* = 3 for Mpc3; error bars indicate standard deviation. **c** Structural view of the TIM9·10 complex [26, 68]. In the chaperone complex (left), Tim9 monomers are shown in dark gray and Tim10 in light gray. Altered amino acids of the mutant variants in the TIM9·10 complex [26] are shown as colored spheres. Tim10 monomer (right) and altered amino acids in the hydrophobic cleft of TIM9·10. **d** Immunoblot of the soluble and insoluble fractions of the cell-free reaction mixtures producing Mpc2 or Mpc3 in the absence of TIM chaperones or in the presence of wild-type TIM9·10 (TIM9·10_WT) or mutant variants of Tim10 in the TIM9·10 complex (TIM9·10_V29K, TIM9·10_F33Q, TIM9·10_M32K, TIM9·10_F70SF33Q). **e** Solubility quantification shows solubility of Mpc2 and Mpc3 in the presence of TIM9·10 mutant variants comparable to the reaction condition without added chaperone complex. *n* = 3; error bars indicate standard deviation; *** and ** indicate the significant difference with *P* < 0.001 and *P* < 0.005, respectively, in comparison with the reaction with the WT chaperone

To directly determine if the MPC precursors depend on the chaperone function of small TIMs, we synthesized cysteine-free forms of the precursors in a cell-free translation system [56] and performed an aggregation assay. The majority of the hydrophobic Mpc2 and Mpc3 precursors aggregated in the cell-free system in the absence of detergent (Fig. 5a, b). Weinhäupl et al. [26] showed that the TIM9·10 chaperone prevented the aggregation of a canonical carrier precursor in vitro. We thus added recombinantly produced TIM9·10 and observed a significant improvement of the solubility of Mpc2 and Mpc3 in a concentration-dependent manner (Fig. 5a, b). Importantly, the positive effect of TIM9·10 on the solubility of MPC precursors was abrogated with Tim10 point mutants in which hydrophobic residues in the binding cleft were replaced by hydrophilic ones (Fig. 5c–e). These mutant forms also disrupt the interaction with carrier precursors [26], suggesting that MPC precursors bind to the same hydrophobic motif of the chaperone as carriers. In addition, we studied the influence of TIM9·10 on Mpc1, whose topology has not been fully clarified but based on a recent homology analysis is likely similar to Mpc2/3, including the lack of a cleavable presequence [6, 7, 36]. We observed a similar prevention of aggregation and dependence on specific Tim10 residues for Mpc1 as for Mpc2 and Mpc3 (Additional file 5: Figure S5a, b). The levels of Mpc1 are considerably reduced in *tim22-14* mitochondria and partially reduced in *tim12-21* and Tim10-L26Q mitochondria (Additional file 3: Figure S3d, Additional file 4: S4d), suggesting that the biogenesis of Mpc1 occurs via the carrier import pathway. Since Mpc1 levels are stable in the absence of Mpc2 and/or Mpc3 (Additional file 1: Figure S1e, f), the observed decrease in *tim22-14*, *tim12-21*, and Tim10-L26Q mitochondria likely reflects a defect in Mpc1 biogenesis rather than an indirect destabilization. In line with our *in organello* import results, the TIM8·13 complex only mildly improved the solubility of MPC precursors (Additional file 5: Figure S5c). We conclude that the TIM9·10 complex chaperones all MPC precursors. Interaction of TIM9·10 with the MPC proteins is mediated by the same conserved Tim10 motifs that are responsible for the chaperone activity toward established substrates [26].

## Discussion

The mitochondrial pyruvate carrier differs substantially from the well-characterized family of mitochondrial carriers, by both its topology and its heterodimeric composition. In particular, all three MPC proteins have their N-termini in the matrix, and for Mpc2 and Mpc3, the presence of three transmembrane helices has been established [6, 7]. Proteins with this topology have been expected to be imported by the TIM23 pathway [14, 15]. In contrast, our results demonstrate that MPC subunits are imported into the inner mitochondrial membrane by the carrier pathway, using all of its characteristic components. They are recognized on the mitochondrial surface by the receptor Tom70, are chaperoned through the intermembrane space by the TIM9·10 complex, and are inserted into the inner membrane by the carrier translocase TIM22. This surprising finding strongly changes the view of the substrate selection of this major transport pathway to the mitochondrial inner membrane.

All studies available so far supported the model that the carrier pathway can only handle pairs of transmembrane helices with their termini in the intermembrane space [18, 19, 23, 25, 26]. Different precursor forms such as truncated carrier precursors or the three-helix Ugo1 are either imported by the highly flexible TIM23 presequence pathway (bypassing the small TIMs), remain in the intermembrane space, or are even directed to the mitochondrial outer membrane [28–33]. The basic requirements of proteins imported by the carrier pathway include paired transmembrane helices with a defined topology, positively charged matrix-exposed segments and the ability to interact with the small TIM chaperones [14, 15, 19, 25–29, 57]. The MPC proteins display a fundamentally different topology but are able to interact with the TIM chaperones, and their matrix-exposed N-termini and loops (between transmembrane helices 2 and 3) are positively charged [1, 2, 7]. The two C-terminal transmembrane helices of Mpc2 and Mpc3 may be handled by the TIM22 machinery similarly to a paired helix of a canonical carrier. The N-terminus of MPCs was suggested to form an amphipathic helix whose function is unknown [7]. As observed for mitochondrial matrix and inner membrane proteins, the matrix-exposed positively charged amino acid residues are likely involved in the translocation of preprotein segments across the inner membrane by responding to Δψ (negative on the matrix side) [24, 25, 57–59]. For the interaction with TIM chaperones, the same residues in the hydrophobic substrate-binding cleft of the TIM9·10 complex are required for the interaction with both types of substrates, MPC precursors and canonical carriers [26], providing strong evidence that the MPCs are bona fide substrates of the carrier import pathway.

## Conclusions

We conclude that the mitochondrial carrier pathway possesses a much higher flexibility than anticipated and can transport transmembrane helices in a paired or non-paired fashion and direct the precursor N-termini into the intermembrane space (canonical carriers, Tim17/22/23) or matrix (MPC proteins). Due to their high conservation, we expect that human MPC subunits [1, 2] are similarly imported into mitochondria via the carrier translocase pathway. These findings represent a striking example that the search for non-canonical substrates can change even long-established views of an essential protein translocation pathway.

## Material and methods

### Yeast strains and growth

The *Saccharomyces cerevisiae* strains used in this study are summarized in Table 1. The strains *tom20*Δ, *tom70*Δ, *tim18*Δ, *tim22-14*, *tim12-21*, *tim17-4*, *tim17-5*, Tim10-L26Q, *tim8*Δ *tim13*Δ, Tim10-L26Q *tim8*Δ *tim13*Δ, *mpc2*Δ *mpc3*Δ, and *mpc1*Δ *mpc2*Δ *mpc3*Δ and their corresponding wild types were described [6, 26, 46, 48, 55, 60–62]. Deletion strains *mpc1*Δ, *mpc2*Δ, and *mpc3*Δ and the corresponding BY4741 wild-type strain were obtained from Euroscarf. Cells for mitochondrial import experiments were grown on YPG media (1% [w/v] yeast extract, 2% [w/v] peptone, 3% [v/v] glycerol) or on YPLac media (1% [w/v] yeast extract, 2% [w/v] peptone, 3% [v/v] glycerol, 0.05% [w/v] CaCl_2_, 0.06% [w/v] MgCl_2_, 0.1% [w/v] KH_2_PO_4_, 0.1% [w/v] NH_4_Cl, 0.05% [w/v] NaCl, 0.05% [w/v] glucose, 2% [v/v] lactate). For the analysis of mitochondrial protein and complex levels in MPC deletion strains, cells were grown on YPG media or on YPD media (1% [w/v] yeast extract, 2% [w/v] peptone, 2% [w/v] glucose). The growth temperature was 30 °C except for the following strains: Tim10-L26Q, *tim8*Δ *tim13*Δ, Tim10-L26Q *tim8*Δ *tim13*Δ, and the corresponding wild-type strain were grown at 21 °C; *tim12-21*, *tim17-4*, *tom20*Δ, and the corresponding wild-type strains were grown at 24 °C; and *tim17-5* and the corresponding wild-type strain were grown at 23 °C.

**Table 1:** *S. cerevisiae* strains used in this study

Strain (lab ID no.)	Genotype	Reference
RL285-16C (SHY WT) (4928)	*MATa his3*Δ*1 ura3Δ0*	[6]
*mpc1*Δ (SHY9) (4929)	*MATa his3*Δ*1 ura3*Δ*0 mpc1::kanMX*	[6]
*mpc2*Δ*mpc3*Δ (SHY14) (4932)	*MATa his3*Δ*1 ura3*Δ*0 mpc2::natMX mpc3::hphMX*	[6]
*mpc1*Δ*mpc2*Δ*mpc3*Δ (SHY15) (4933)	*MATa his3*Δ*1 ura3*Δ*0 mpc1::kanMX mpc2::natMX mpc3::hphMX*	[6]
YPH499 (WT) (1501)	*MATa ura3-52 lys2-801 ade2-101 trp1-*Δ*63 his3-*Δ*200 leu2-*Δ*1*	[63]
*tom20*Δ (1273)	*MATa ura3-52 lys2-801 ade2-101 trp1-*Δ*63 his3-*Δ*200 leu2-*Δ*1 tom20::URA3 pYEP-TOM22*	[62]
*tom70*Δ (1183)	*ura3-52 lys2-801 ade2-101 trp1-*Δ*63 his3-*Δ*200 leu2-*Δ*1 tom70::HIS3*	[60, 61]
*tim18*Δ (1383)	*MATa ura3-52 lys2-801 ade2-101 trp1-*Δ*63 his3-*Δ*200 leu2-*Δ*1 tim18::kanMX6*	[48]
*tim22-14* (1370) (YPH499 22-M4)	*MATa ura3-52 lys2-801 ade2-101 trp1-*Δ*63 his3-*Δ*200 leu2-*Δ*1 tim22-M4* (amino acid alterations in Tim22-14: I11M, K16R, E21K, G31R, N37D, F63L, A85T, T86A, K120R, C141S, Y153H, M193 T, K194Q)	[48], this study
*tim12-21* (2462) (YPH-BG-12-1)	*MATa ura3-52 lys2-801 ade2-101 trp1-*Δ*63 his3-*Δ*200 leu2-*Δ*1 tim12::ADE2 pFL39-TIM12-1ts* (amino acid alterations in Tim12-21: S7G, V14D, A22E, D64A)	[55]
*tim17-4* (1758) (YPH-BG17-9d)	*MATa ura3-52 lys2-801 ade2-101 trp1-*Δ*63 his3-*Δ*200 leu2-*Δ*1 BG17-9d (tim17-4)* (amino acid alteration in Tim17-4: C10R)	[46, 47], this study
*tim17-5* (1759) (YPH-BG17-21-7)	*MATa ura3-52 lys2-801 ade2-101 trp1-*Δ*63 his3-*Δ*200 leu2-*Δ*1 BG17-21-7 (tim17-5)* (amino acid alterations in Tim17-5: P42L, R109G, S115P)	[46, 47], this study
WT for Tim10 mutants (5118)	*MATa ura3-52 lys2-801 ade2-101 trp1-*Δ*63 his3-*Δ*200 leu2-*Δ*1 tim10::ADE2 pFL39-TIM10*	[26]
Tim10-L26Q (5210)	*MATa ura3-52 lys2-801 ade2-101 trp1-*Δ*63 his3-*Δ*200 leu2-*Δ*1 tim10::ADE2 pFL39-TIM10-L26Q*	[26]
*tim8*Δ*tim13*Δ (5084)	*MATa ura3-52 lys2-801 ade2-101 trp1-*Δ*63 his3-*Δ*200 leu2-*Δ*1 tim8::natNT2 tim13::hphNT1 tim10::ADE2 pFL39-TIM10*	[26]
Tim10-L26Q *tim8*Δ*tim13*Δ (5206)	*MATa ura3-52 lys2-801 ade2-101 trp1-*Δ*63 his3-*Δ*200 leu2-*Δ*1 tim8::natNT2 tim13::hphNT1 tim10::ADE2 pFL39-TIM10-L26Q*	[26]
BY4741 (WT) (1354)	*MATa ura3*Δ*0 his3*Δ*1 leu2*Δ*0 met15*Δ*0*	Euroscarf
*mpc1*Δ (4774)	*MATa ura3*Δ*0 his3*Δ*1 leu2*Δ*0 met15*Δ*0 mpc1::kanMX4*	Euroscarf
*mpc2*Δ (4775)	*MATa ura3*Δ*0 his3*Δ*1 leu2*Δ*0 met15*Δ*0 mpc2::kanMX4*	Euroscarf
*mpc3*Δ (4776)	*MATa ura3*Δ*0 his3*Δ*1 leu2*Δ*0 met15*Δ*0 mpc3::kanMX4*	Euroscarf

### Isolation of mitochondria

Mitochondria were isolated by fractionation [64]. After pre-treatment with DTT buffer (100 mM Tris-H_2_SO_4_ pH 9.4, 10 mM DTT) and digestion of the cell wall with zymolyase in zymolyase buffer (20 mM potassium phosphate buffer pH 7.4, 1.2 M sorbitol), the cells were lysed on ice in homogenization buffer (10 mM Tris-HCl pH 7.4, 0.6 M sorbitol, 1 mM EDTA, 0.2% bovine serum albumin, 1 mM phenylmethylsulfonyl fluoride (PMSF)) with a glass Teflon homogenizer. After two centrifugation steps at 2000×*g* to remove the cell debris and nuclei, crude mitochondria were isolated from the supernatant by centrifugation at 17,000×*g*. Mitochondria were resuspended in SEM buffer (250 mM sucrose, 1 mM EDTA, 10 mM MOPS-KOH pH 7.2) and stored at − 80 °C.

### *In organello* import

In vitro synthesis of [^35^S]methionine-labeled precursor proteins was performed with the mMessage mMachine SP6 transcription kit (Ambion, Cat.# 1340) and the Flexi rabbit reticulocyte translation kit (Promega, Cat. # L4540), or with the TNT SP6 coupled reticulocyte transcription/translation kit (Promega, Cat. # L2080). The following plasmids were used as templates: pGEM4Z-AAC (*Neurospora crassa*), pGEM-F_1_β (*S. cerevisiae*), pGEM4Z-b_2_(220)-DHFR, pGEM4Z-Mpc1, pGEM4Z-Mpc2, and pGEM4Z-Mpc3. The radiolabeled precursors were imported into the isolated mitochondria at 25 °C in import buffer (10 mM MOPS-KOH pH 7.2, 3% [w/v] bovine serum albumin, 250 mM sucrose, 80 mM KCl, 5 mM MgCl_2_, 2 mM KH_2_PO_4_, 5 mM methionine) with 2–4 mM NADH and an ATP-regenerating system including 2–4 mM ATP, 5–10 mM creatine phosphate, and 0.1–0.2 mg/ml creatine kinase. Import reactions into *tim12-21* and the control wild-type mitochondria were performed at 30 °C. *tim17-4* mitochondria and *tim17-5* mitochondria and the corresponding wild-type mitochondria were heat-shocked for 10 min at 37 °C in import buffer prior to the addition of NADH, the ATP-regenerating system, and the radiolabeled precursor proteins (in reticulocyte lysate), followed by the import reaction at 25 °C. Reactions included a control sample where the membrane potential was dissipated with AVO mix (8 μM antimycin A, 1 μM valinomycin, 20 μM oligomycin) before the addition of precursor. The import reactions were stopped by the addition of AVO and transfer on ice. Non-imported precursor was removed by a 15-min incubation with 50 μg/ml proteinase K on ice, unless indicated otherwise. After the inactivation of proteinase K with 2 mM PMSF, the mitochondria were reisolated and washed in SEM buffer. To generate mitoplasts after the import reaction, the mitochondria were resuspended in hypotonic EM buffer (1 mM EDTA, 10 mM MOPS-KOH pH 7.2). The mitoplasts were treated with 50 μg/ml proteinase K and subsequently treated with PMSF and re-isolated as described above. Quantification of import/assembly efficiency was performed with Fiji ImageJ software. Replicates used for quantification were independent import and assembly assays of incubation of isolated yeast mitochondria (wild-type and mutant mitochondria) with radiolabeled precursor proteins, followed by independent gel separation and analysis. The individual data values from independent replicates are listed in Additional file 6: Table S1 and Additional file 7: Table S2.

### Gel electrophoresis and Western blotting

Import reactions were analyzed by SDS-PAGE or blue native gel electrophoresis (BN-PAGE) and autoradiography. For BN-PAGE analysis [65], mitochondria were solubilized in solubilization buffer (20 mM Tris-HCl pH 7.4, 50 mM NaCl, 0.1 mM EDTA, 10% [v/v] glycerol, 1% [w/v] digitonin, 1 mM PMSF) or in low-ionic strength buffer (50 mM imidazole-HCl pH 7.0, 500 mM 6-aminohexanoic acid, 1 mM EDTA, 3% [w/v] digitonin, 1 mm PMSF) [66] for 15 min on ice. Analysis of protein levels and native protein complexes was performed by SDS-PAGE or BN-PAGE, respectively, followed by Western blot analysis. The following rabbit antisera were used (source: Pfanner Lab, non-commercial antisera specifically prepared for the lab): α-Mpc1 (GR5021-1, 1:100), α-Mpc2 (GR5024-4, affinity purified, 1:100), α-Mpc3 (GR5025-5, affinity purified, 1:100), α-Tim22 (GR5113-4, 1:250), α-Tim54 (GR2012-3, 1:200), α-Tim18 (GR5114-3, 1:250), α-Tim12 (GR905-1, 1:500), α-Yhm2 (GR3053-5, 1:500), α-Ssc1 (GR1830-7, 1:250), α-Tom70 (GR657-5, 1:500), α-Tom40 (168-12/5, 1:500), α-Tom20 (GR3225-7, 1:5000), α-Tim23 (133-6, 1:500), α-Tim17 (GR1844-4, 1:500), α-Cor1 (GR371-6, 1:500), α-Tim13 (GR2044-5, 1:500), α-Tim10 (GR2041-7, 1:250), and α-Atp4 (GR1958-4, 1:500). α-rabbit IgG-peroxidase was obtained from Sigma-Aldrich (A6154, 1:5000–1:10,000).

### Cell-free expression of MPC proteins in the absence or presence of TIM chaperones

Genes coding for *S. cerevisiae* Mpc1(C87A), Mpc2(C86A, C111S), and Mpc3(C87A) were cloned by GeneCust in customized pIVEX2.3d cell-free expression plasmids between NdeI and XhoI cloning sites. Cysteine-free variants were used since previous studies with the chaperoning assay [26] indicated that the presence of Cys residues can lead to enhanced aggregation, likely due to disulfide formation. The plasmid codes for the TEV-protease-cleavable N-terminal His_6_-tag, and it includes the stop codon before the C-terminal His_6_-tag of the original plasmid. The produced MPC proteins contain a cleavable His_6_-tag at the N-terminus.

MPC proteins were produced in 50 μl cell-free reaction mixtures [67] for 2.5 h at 28 °C. The final composition of the cell-free reaction buffer was 0.08 mM rUTP, 0.08 mM rGTP, 0.08 mM rCTP, 0.55 mM HEPES, 0.12 mM ATP, 6.8 μM folinic acid, 0.064 mM cyclic AMP, 0.34 mM DTT, 2.75 mM NH_4_OAc, 80 mM phosphocreatine, 0.208 M potassium glutamate, 10.48 mM magnesium acetate, 1 mM of amino acid mix, 1.25 μg creatine kinase, 0.25 μg T7 polymerase, 20 μl S30 *E. coli* extract, 0.5 μg plasmid DNA, and 0.175 mg/ml tRNAs. The reaction condition with the detergent contained additionally 0.5% of Brij35. To test the specificity of the binding of MPC proteins by TIM chaperones, the solubility of MPC proteins was monitored at increasing concentration of either TIM8·13 or TIM9·10 complexes. The concentrations of the chaperones in the reaction mixtures were 0, 0.2, 1, 2, and 4 mg/ml. To test the effect of selected Tim10 mutant variants in the TIM9·10 chaperone complex on the binding and subsequently the solubility of MPC proteins, 4 mg/ml of the TIM9·10_WT, TIM9·10_V29K, TIM9·10_F33Q, TIM9·10_M32K, and TIM9·10_F70SF33Q were used. Chaperone complexes of TIM8·13, TIM9·10, and mutant variants of TIM9·10 for cell-free experiments were expressed and purified as described previously [26].

The cell-free reaction was stopped after 2.5 h, and the soluble fraction was separated from the insoluble pellet by centrifugation at 16.800×*g*. The amount of His-tagged MPC proteins in the soluble fraction and the pellet were quantified from the membranes, after the immunodecoration with anti-His antibody (Sigma-Aldrich monoclonal α-polyHistidine-peroxidase antibody; product no: A7058), as relative band intensities using BioRad ImageLab program/software. The solubility of the proteins was calculated from at least three experiments for each condition, as a percentage of protein in the supernatant in relation to the total amount of protein in the pellet and supernatant. Significance of the difference in solubility between wild-type TIM9·10 and the mutant variants was analyzed with GraphPad Prism 5 using one-way ANOVA and Tukey’s multiple comparison test. The individual data values from independent replicates are listed in Additional file 6: Table S1 and in Additional file 7: Table S2.

## Supplementary information

**Additional file 1:** **Figure S1.** Assembly and level of MPC subunits. (PDF)

**Additional file 2:** **Figure S2.** Characterization of mitochondria lacking Tom20 or Tom70. (PDF)

**Additional file 3:** **Figure S3.** Characterization of mitochondria affected in TIM23 or TIM22 translocases. (PDF)

**Additional file 4:** **Figure S4.** Characterization of mitochondria affected in small TIM chaperones. (PDF)

**Additional file 5:** **Figure S5.** Interaction of Mpc1, Mpc2 and Mpc3 with TIM chaperones in vitro. (PDF)

**Additional file 6:** **Table S1.** Individual data values for quantifications in main figures. (XSLX)

**Additional file 7:** **Table S2.** Individual data values for quantifications in supplementary figures. (XSLX)

## Data Availability

All data generated or analyzed during this study are included in this published article and its supplementary data (Additional files 1, 2, 3, 4, 5, 6, and 7).
