# Supplementary material for: The mitochondrial carrier pathway transports non-canonical substrates with an odd number of transmembrane segments
Source: BMC Biol. 2020 Jan 6;18:2. doi: 10.1186/s12915-019-0733-6 (PMC6945462; doi:10.1186/s12915-019-0733-6)
Supplement: Supplementary file 1 — Additional file 1: Figure S1. Assembly and level of MPC subunits. (PDF) [file 12915_2019_733_MOESM1_ESM.pdf]

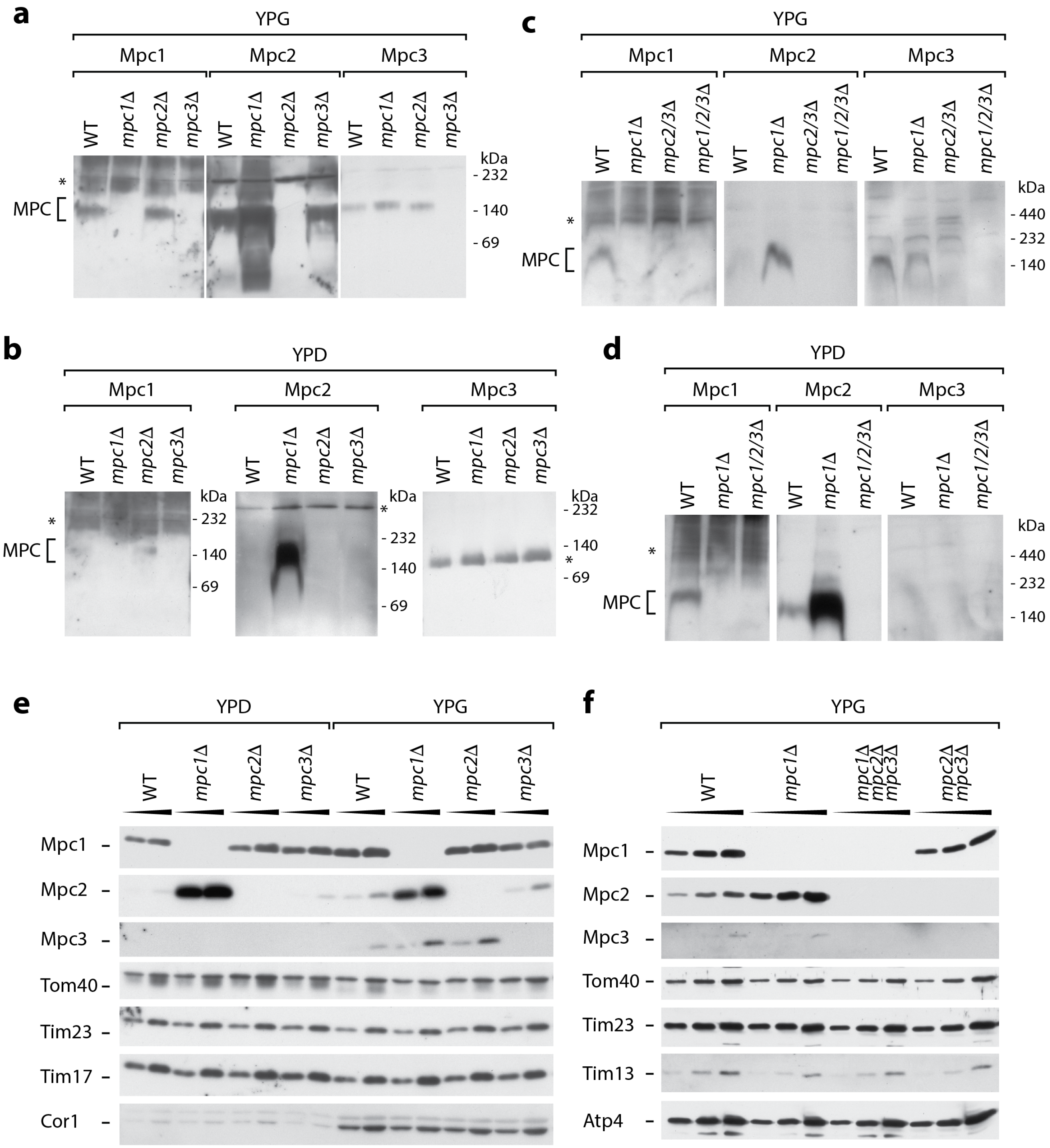

Fig. S1. Assembly and levels of MPC subunits. Mitochondria were isolated from the indicated *S. cerevisiae* strains. MPC complexes were analyzed by BN-PAGE and Western blotting (**a-d**). Protein levels of MPC subunits and control proteins were analyzed by SDS-PAGE and Western blotting (**e-f**). Yeast strains of BY4741 background (**a-b, e**) and of RL285-16C background (**c-d, f**) [6] were grown on respiratory YPG media (**a, c, e-f**) or fermentative YPD media (**b, d, e**) prior to mitochondria isolation. Asterisk: non-specific band. MPCs function as heterodimers,

Mpc1-Mpc2 and Mpc1-Mpc3 [1,2,6,7,13]. However, MPC subunits are also known to form non-functional homodimers with similar complex sizes [6,7|, seen e.g. in (**a)** with the Mpc3 complex in *mpc1*Δ mitochondria that migrates only slightly more slowly than the Mpc1-Mpc3 heterodimer seen in WT and *mpc2*Δ mitochondria. The relative prevalence of MPC homodimers and their possible crosstalk with other MPC subunits have not been clarified. The steady-state levels of Mpc2 and Mpc2 complex formation were strongly increased in the absence of Mpc1 (seen in **a-f**) [1].
