# Supplementary material for: The mitochondrial carrier pathway transports non-canonical substrates with an odd number of transmembrane segments
Source: BMC Biol. 2020 Jan 6;18:2. doi: 10.1186/s12915-019-0733-6 (PMC6945462; doi:10.1186/s12915-019-0733-6)
Supplement: Supplementary file 2 — Additional file 2: Figure S2. Characterization of mitochondria lacking Tom20 or Tom70. (PDF) [file 12915_2019_733_MOESM2_ESM.pdf]

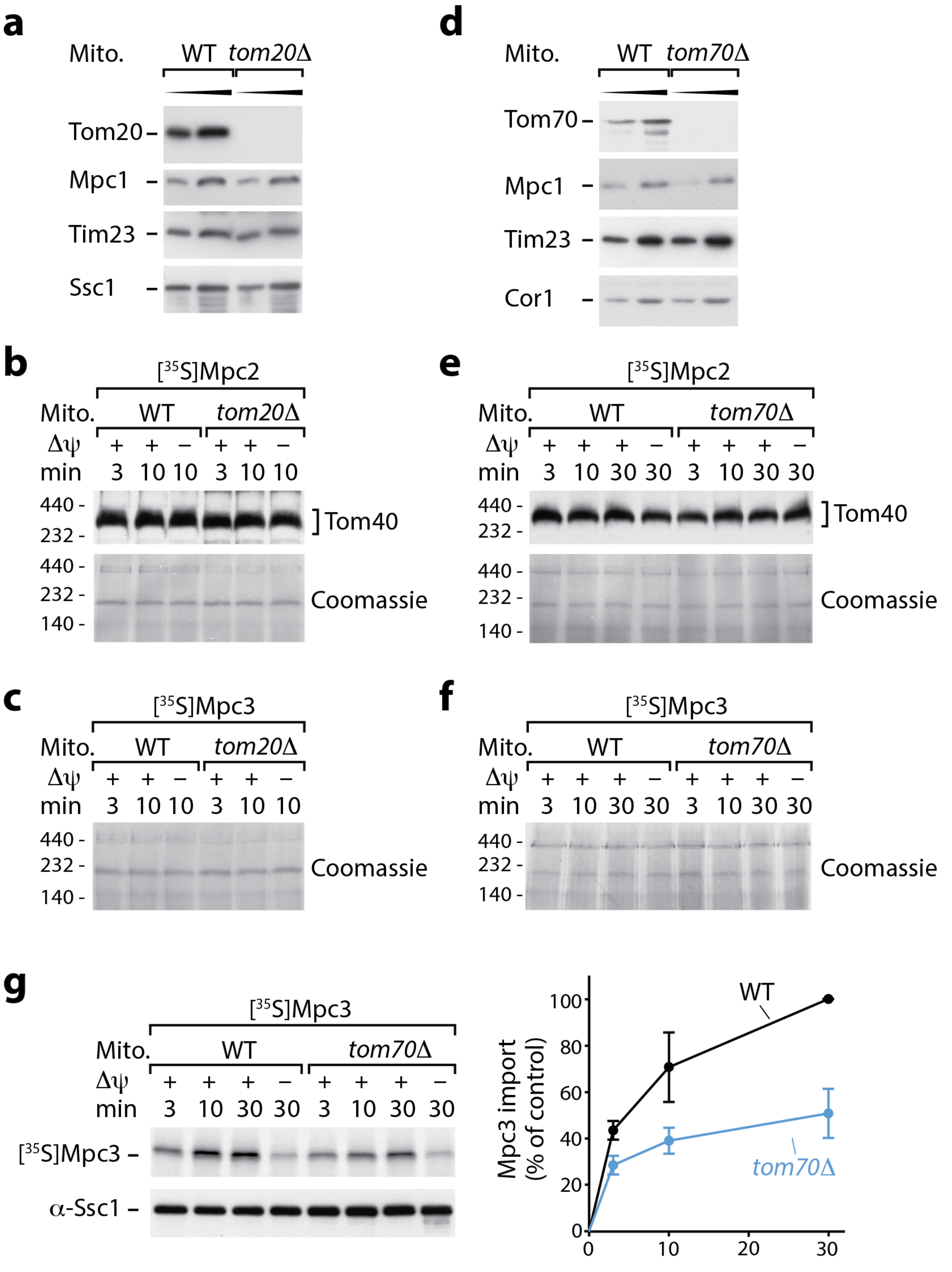

Fig. S2. Characterization of mitochondria lacking Tom20 or Tom70. **a** Protein levels of the mitochondria lacking Tom20 (10 and 25 μg total mitochondrial protein) were analyzed by SDS-PAGE and Western blotting. Tim23, component of the TIM23 translocase; Ssc1, mitochondrial Hsp70, component of PAM. **b-c** Loading controls. Radiolabeled Mpc2 and Mpc3 were imported into mitochondria lacking Tom20 as described in Fig. 2a-b, import reactions were analyzed by BN-PAGE and Western blotting, and immunodecorated for the TOM complex (α-Tom40) or stained with

Coomassie Blue R-250 to control for equal loading. Representative import experiments are shown. **d** Protein levels of the mitochondria lacking Tom70 (15 and 30 μg total mitochondrial protein) were analyzed by SDS-PAGE analysis and Western blotting. Tim23, component of the TIM23 translocase; Cor1, component of complex III of the respiratory chain. **e-f** Loading controls. Radiolabeled Mpc2 and Mpc3 were imported into mitochondria lacking Tom70 as described in Fig. 2a-b, import reactions were analyzed by BN-PAGE and Western blotting, and immunodecorated for the TOM complex (α-Tom40) or stained with Coomassie to control for equal loading. Representative import experiments are shown. **g** Radiolabeled Mpc3 was imported into wildtype or *tom70*Δ mitochondria as described in Fig. 2b and import efficiency was analyzed by SDS-PAGE, Western blotting and autoradiography (upper panel). Equal loading was controlled by immunodecoration against Ssc1 (lower panel). Right panel: Quantification of Mpc3 import efficiency; the efficiency into WT mitochondria after 30 min was set to 100% (control); n = 3; error bars: SEM. In all import experiments, non-imported precursors were degraded with proteinase K.
