# Supplementary material for: The mitochondrial carrier pathway transports non-canonical substrates with an odd number of transmembrane segments
Source: BMC Biol. 2020 Jan 6;18:2. doi: 10.1186/s12915-019-0733-6 (PMC6945462; doi:10.1186/s12915-019-0733-6)
Supplement: Supplementary file 3 — Additional file 3: Figure S3. Characterization of mitochondria affected in TIM23 or TIM22 translocases. (PDF) [file 12915_2019_733_MOESM3_ESM.pdf]

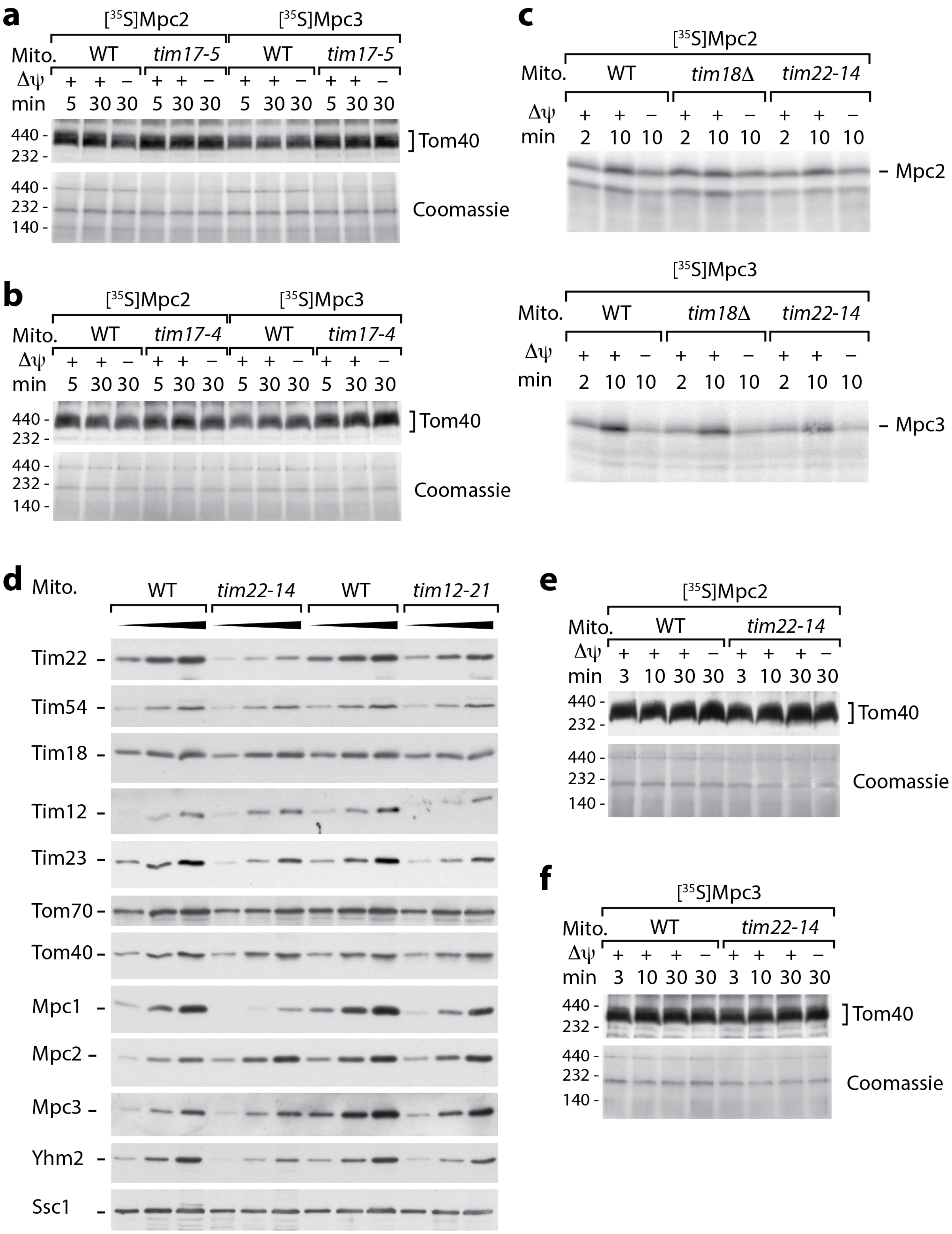

Fig. S3. Characterization of mitochondria affected in TIM23 or TIM22 translocases. **a-b** Loading controls. Radiolabeled Mpc2 and Mpc3 were imported into mitochondria isolated from the indicated strains as described in Fig. 3a+c, import reactions were analyzed by BN-PAGE and Western blotting, and immunodecorated for the TOM

complex (α-Tom40) or stained with Coomassie to control for equal loading. Representative import experiments are shown. **c** Radiolabeled Mpc2 and Mpc3 were imported into wild-type, *tim18Δ* or *tim22-14* mitochondria for the indicated periods. Mitoplasts were generated by hypo-osmotic swelling and treated with proteinase K. The samples were analyzed by SDS-PAGE and autoradiography. In all import experiments, non-imported precursors were degraded with proteinase K. **d** Steady-state protein levels of TIM22 mutant mitochondria. Mitochondria (10, 20 and 40 μg total mitochondrial protein) isolated from wild-type, *tim22-14* or *tim12-21* yeast strains were analyzed by SDS-PAGE and Western blotting with the indicated antisera. Tim22, Tim54, Tim18, Tim12, TIM22 components; Tim23, TIM23 translocase component; Yhm2, citrate/oxoglutarate carrier (canonical mitochondrial carrier); Ssc1, mitochondrial Hsp70, component of PAM; Tom70, Tom40, components of the TOM translocase. **e-f** Loading controls. Radiolabeled Mpc3 was imported into mitochondria isolated from the indicated strains as described in Fig. 3e+f, import reactions were analyzed by BN-PAGE and Western blotting, and immunodecorated for the TOM complex (α-Tom40) or stained with Coomassie to control for equal loading. Representative import experiments are shown.
