# Supplementary material for: The mitochondrial carrier pathway transports non-canonical substrates with an odd number of transmembrane segments
Source: BMC Biol. 2020 Jan 6;18:2. doi: 10.1186/s12915-019-0733-6 (PMC6945462; doi:10.1186/s12915-019-0733-6)
Supplement: Supplementary file 4 — Additional file 4: Figure S4. Characterization of mitochondria affected in small TIM chaperones. (PDF) [file 12915_2019_733_MOESM4_ESM.pdf]

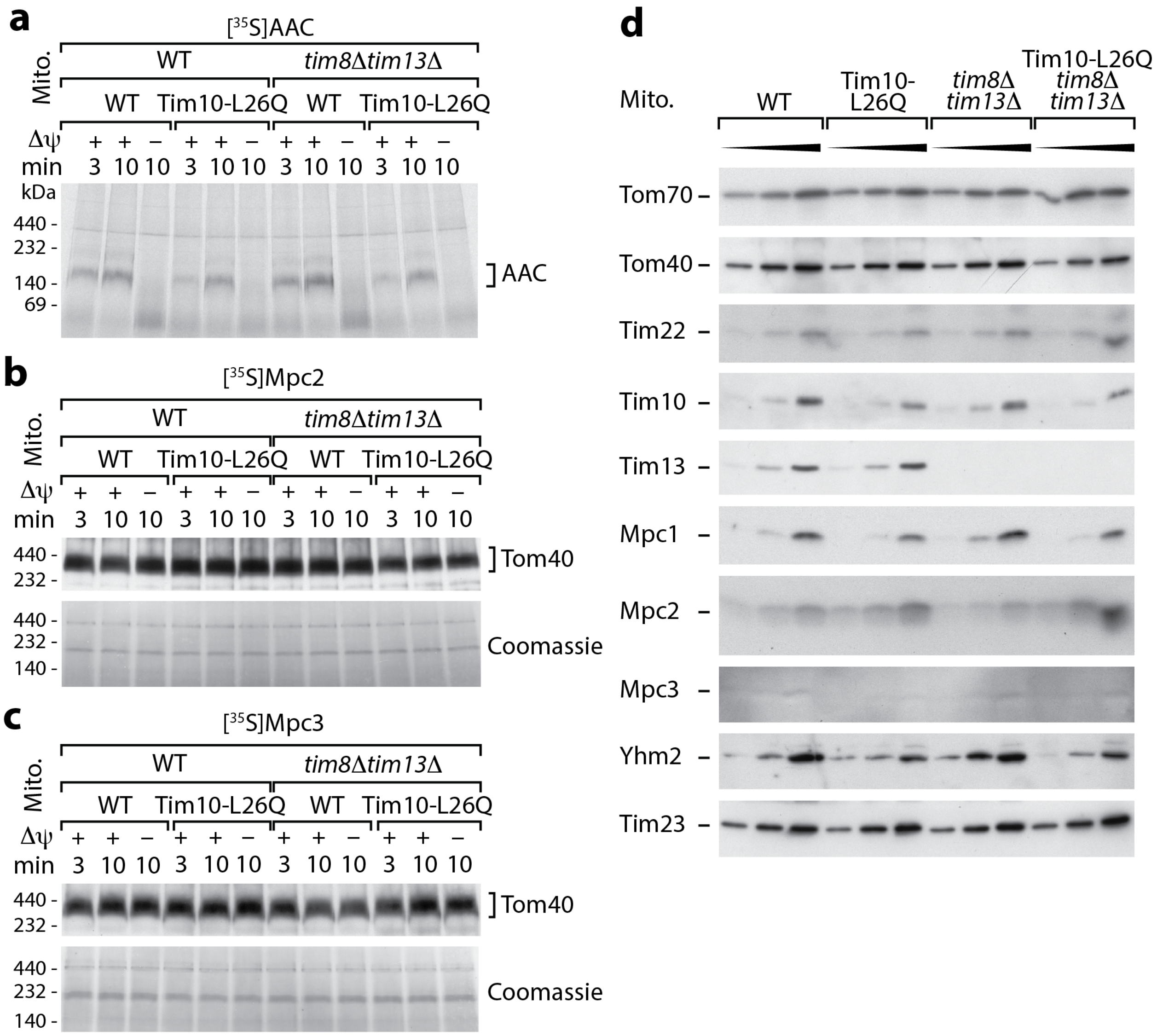

Fig. S4. Characterization of mitochondria affected in small TIM chaperones. **a** The carrier protein AAC was imported into WT mitochondria, mitochondria with the mutant form Tim10-L26Q, mitochondria lacking Tim8 and Tim13, or mitochondria affected in Tim10, Tim8 and Tim13, and samples were analyzed by BN-PAGE and autoradiography as described in Fig. 4a. **b-c** Loading controls. Radiolabeled Mpc2 or Mpc3 were imported as described in Fig. 4a into mitochondria isolated from the indicated strains, import reactions were analyzed by BN-PAGE and Western blotting, and immunodecorated for the TOM complex (α-Tom40) or stained with Coomassie to control for equal loading. Representative import experiments are shown. In all import experiments, non-imported precursors were degraded with proteinase K. **d** Steady-state protein levels of mitochondria from the indicated strains. Mitochondria (10, 20 and 40 μg total mitochondrial protein) were analyzed by SDS-PAGE and Western

blotting with the indicated antisera. Tom70, Tom40, components of the TOM translocase; Tim22, Tim10, Tim13, TIM22 pathway components; Yhm2, citrate/oxoglutarate carrier (canonical mitochondrial carrier); Tim23, component of the TIM23 translocase.
