# Supplementary material for: The mitochondrial carrier pathway transports non-canonical substrates with an odd number of transmembrane segments
Source: BMC Biol. 2020 Jan 6;18:2. doi: 10.1186/s12915-019-0733-6 (PMC6945462; doi:10.1186/s12915-019-0733-6)
Supplement: Supplementary file 5 — Additional file 5: Figure S5. Interaction of Mpc1, Mpc2 and Mpc3 with TIM chaperones in vitro. (PDF) [file 12915_2019_733_MOESM5_ESM.pdf]

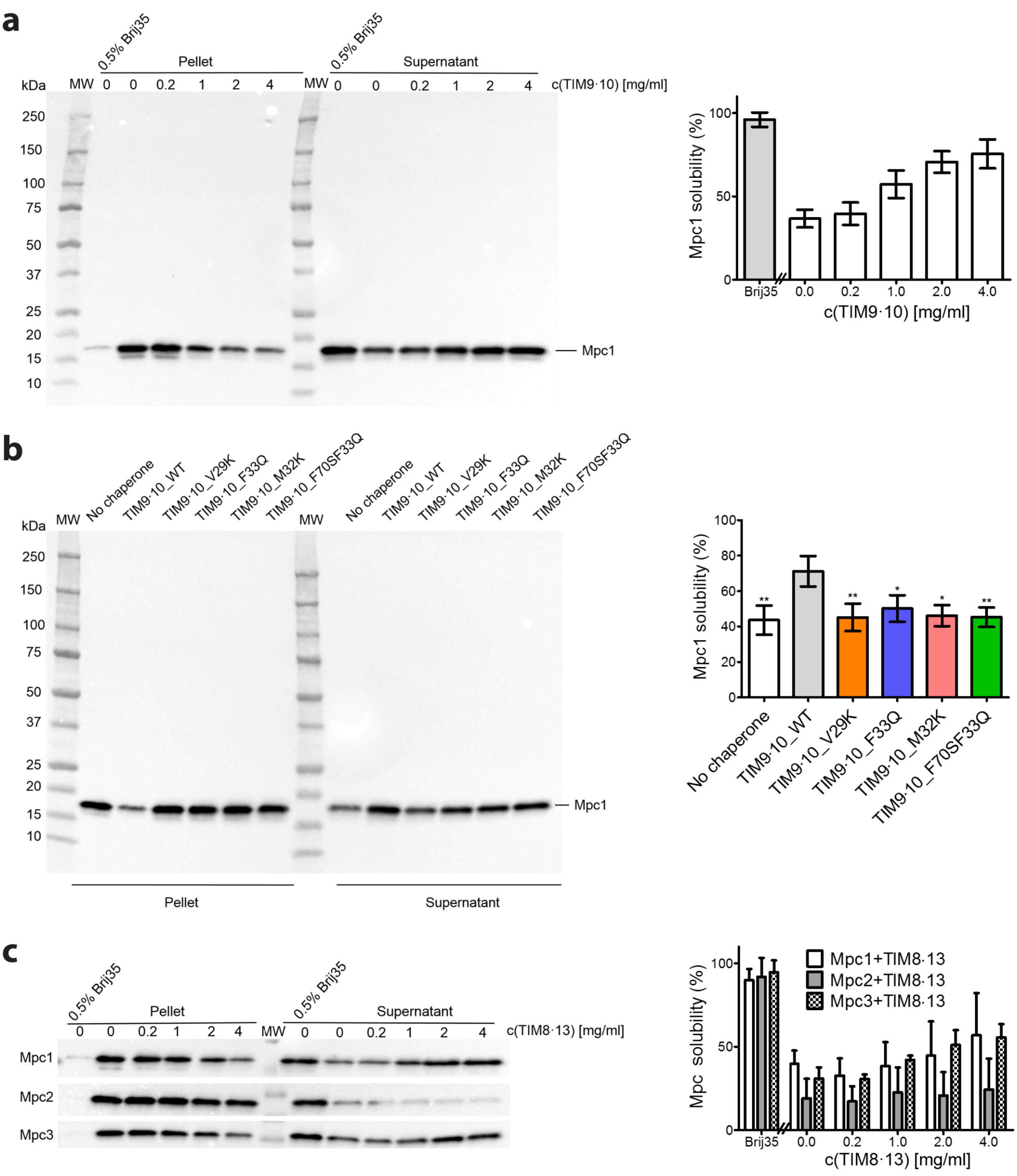

Fig. S5. Interaction of Mpc1, Mpc2 and Mpc3 with TIM chaperones *in vitro*. **a** Production of Mpc1 in cell-free reaction mixtures supplemented with detergent (Brij35) or different concentrations of recombinantly produced TIM9·10 chaperone complex. Immunoblot (left) of the soluble (supernatant) and insoluble (pellet) fractions of the cell-free reaction mixtures. Mpc1 solubility quantification (right). n = 3; error bars indicate standard deviation. In the presence of detergent (absence of TIM9·10), Mpc1 was mostly found in the soluble fraction. In the absence of detergent and chaperone,

the majority of Mpc1 was found in the insoluble fraction. Increasing the concentration of TIM9·10 chaperone complex in the reaction mixture resulted in increased solubility of Mpc1. **b** Immunoblot (left) of the soluble and insoluble fractions of the cell-free reaction mixtures producing Mpc1 in the absence of TIM chaperones or in the presence of wild-type or mutant variants of Tim10 in the TIM9·10 complex. Mpc1 solubility quantification (right) shows solubility of Mpc1 in the presence of mutant variants of the chaperone comparable to the reaction condition without added chaperone complex. n = 3; error bars indicate standard deviation; ** and * indicate the significant difference with $P < 0.005$ or $P < 0.05$, respectively, in comparison to the reaction with the WT chaperone. **c** Immunoblot (left) of the soluble and insoluble fractions of the cell-free reaction mixtures producing Mpc1, Mpc2 or Mpc3, supplemented with detergent (Brij35) or different concentrations of recombinantly produced TIM8·13 chaperone complex. Quantification of the solubility of the Mpc precursors (right). n = 3; error bars indicate standard deviation. In the presence of detergent, MPC precursors were mostly found in the soluble fraction. In the absence of detergent and chaperone, the majority of MPC precursors was found in the insoluble fraction. TIM8·13 exerted a lower chaperoning activity toward MPC precursors than TIM9·10.
